# Delayed hyper-enhancement cardiac magnetic resonance provides incremental prognostic value in patients with cardiac amyloidosis

**DOI:** 10.1186/1532-429X-11-S1-O65

**Published:** 2009-01-28

**Authors:** Bethany A Austin, Scott Flamm, E Rene Rodriguez, Carmela Tan, WH Wilson Tang, David O Taylor, Randall C Starling, Milind Y Desai

**Affiliations:** Cleveland Clinic, Cleveland, OH USA

**Keywords:** Cardiac Magnetic Resonance, Left Atrial, High Diagnostic Accuracy, Cardiac Amyloidosis, Myocardial Performance Index

## Introduction

Patients with cardiac amyloidosis (CA) have an unfavorable, albeit a variable prognosis. In patients with documented cardiac amyloidosis (CA), delayed hyper-enhancement-cardiac magnetic resonance (DHE-CMR) has been demonstrated to have a high diagnostic accuracy. However, its prognostic utility in CA has not been determined.

## Purpose

We sought to determine the incremental prognostic value of DHE-CMR in CA.

## Methods

We studied 47 consecutive patients with suspected CA (mean age 63 ± 13 years, 70% men, 55% with NYHA class > 2) that underwent electrocardiography (ECG), transthoracic echocardiography (TTE), DHE-CMR (Siemens 1.5 T scanner, Erlangen, Germany) and biopsy (38 endomyocardial, 9 extracardiac) between 1/05 and 7/08. Low voltage on ECG was defined as sum of S wave in lead V1 + R wave in lead V5 or V6 < 15 mm. Measured TTE parameters included left atrial size, interventricular septal thickness, speckled appearance, E/A ratio, E/E' ratio, stage of diastology, deceleration time (msec) and myocardial performance index [(isovolumic contraction time + isovolumic relaxation time)/ejection time]. DHE-CMR images were obtained in standard long and short axis orientations (covering the entire LV), after injection of Gadolinium dimenglumine using an inversion recovery spoiled gradient echo sequence: TE 4 msec, TR 8 msec, flip angle 30°, bandwidth 140 Hz/pixel, 23 k-space lines acquired every other RR-interval, field of view (varied from 228–330 in the x-direction and 260–330 in the y-direction) and matrix size (varied from 140–180 in the x-direction and 256 in the y-direction). CMR was considered positive in the presence of DHE of entire subendocardium with extension into the neighboring myocardium. All-cause mortality was ascertained.

## Results

**Figure 1:**
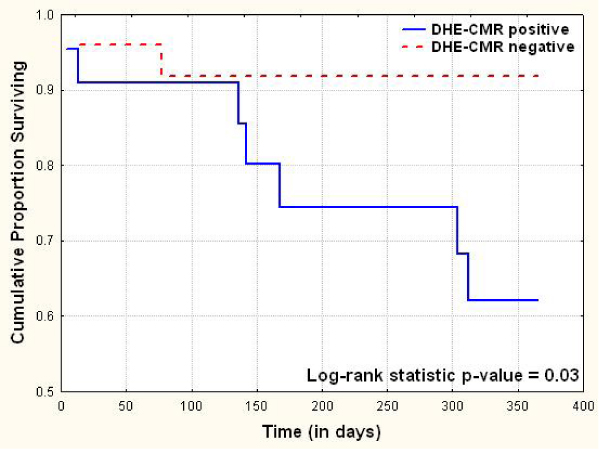
Figure 1

At baseline, 59% patients had low voltage on ECG, while 67% had deceleration time < 150 msec and 53% had E/E' > 15 (both on Doppler echocardiography). Mean MPI, left ventricular ejection fraction and interventricular septal thickness were 0.51 ± 0.3, 51% ± 13 and 1.5 cm ± 0.5, respectively. At up to 1-year after biopsy, there were 9 (19%) deaths. Results of Cox Proportional Hazard survival analysis are shown in Table 1. On univariate Kaplan-Meier survival analysis, presence of DHE on CMR was associated with worse 1-year survival (log rank statistic p-value = 0.03, Figure 1).

**Table 1:** Cox proportional hazard analysis of various clinical and noninvasive imaging predictors of long-term mortality in patients with biopsy proven cardiac amyloidosis

	Univariate Analysis		Multivariate Analysis
Variable	χ^2^	p value	p value
Age	3.8	0.05	0.10
Gender	0.15	0.69	
New York Heart Association Class	3.3	0.07	0.16
Low voltage on electrocardiogram	0.67	0.41	
Left atrial size > 20 cm2	0.76	0.39	
Left ventricular ejection fraction	2.21	0.14	
Interventricular septal thickness	1.7	0.19	
E/E' > 15 on Doppler echocardiography	1.12	0.29	
Deceleration time on Doppler echocardiography ≤ 150 msec	1.41	0.23	
Myocardial performance index	2.10	0.15	
Diastology grade	0.35	0.55	
Delayed hyperenhancement on cardiac magnetic resonance	4.91	0.03	0.02

Chi-square for multivariate model = 12.27, p-value = 0.007

## Conclusion

Presence of DHE on CMR is associated with worse 1-year survival in CA. Along with a high diagnostic accuracy; DHE-CMR adds incremental prognostic value in CA, independent of other variables.

